# Ultrafast Polarization Switching in BaTiO_3_ Nanomaterials: Combined Density Functional Theory and Coupled Oscillator Study

**DOI:** 10.1021/acsomega.3c07741

**Published:** 2024-01-17

**Authors:** Petr Zhilyaev, Kirill Brekhov, Elena Mishina, Christian Tantardini

**Affiliations:** † MIREA—Russian Technological University, Vernadsky Avenue 78, Moscow 119454, Russia; ‡ Hylleraas Center, UiT the Arctic University of Norway, P.O. Box 6050 Langnes, Tromsø N-9037, Norway; § Department of Materials Science and NanoEngineering, Rice University, Houston, Texas 77005, United States of America; ∥ Institute of Solid State Chemistry and Mechanochemistry SB RAS, ul. Kutateladze 18, Novosibirsk 630128, Russian Federation

## Abstract

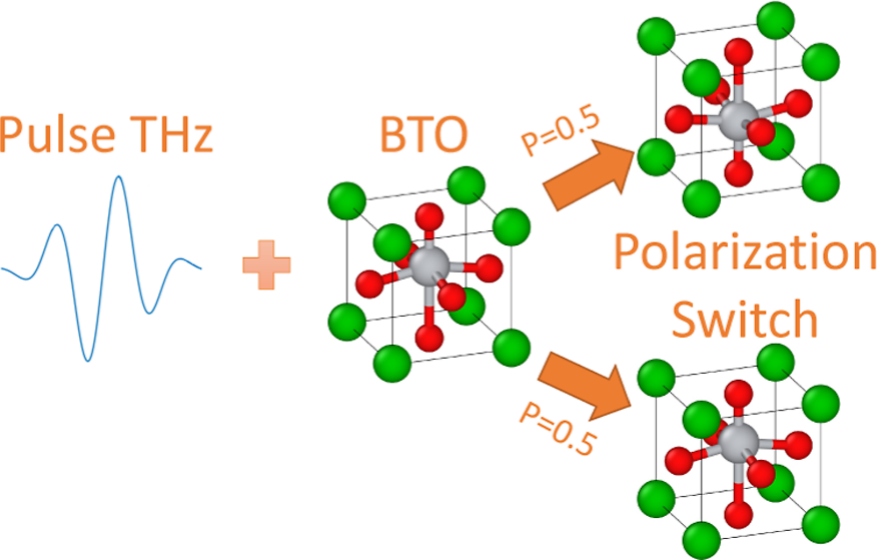

The challenge of achieving ultrafast switching of electric polarization in ferroelectric materials remains unsolved as there is no experimental evidence of such switching to date. In this study, we developed an enhanced model that describes switching within a two-dimensional space of generalized coordinates at THz pulses. Our findings indicate that stable switching in barium titanate cannot be achieved through a single linearly polarized pulse. When the intensity of the linearly polarized pulse reaches a certain threshold, the sample experiences depolarization but not stable switching. Our study also reveals that phonon friction plays a minor role in the switching dynamics and provides an estimate of the optimal parameters for the perturbing pulse with the lowest intensity that results in the depolarization of an initially polarized sample.

## Introduction

Developing nonvolatile memory devices with fast writing and reading operations while minimizing power consumption is a challenge in information storage. However, traditional magnetic storage and flash may not be suitable for future fast devices due to their limited operation speed, which is in the milliseconds range. Thus, this challenge can be addressed only by utilizing different physical mechanisms for writing and reading bits. A potential physical mechanism for write operation is magnetization switching by an ultrashort electromagnetic pulse of optical or THz range. This mechanism has been shown promising in previous studies.^1−3^ Similarly, electric fields can be utilized for ultrafast polarization switching in ferroelectric materials. Although this possibility has garnered significant attention, it has not yet been observed experimentally. The closest successful result to date, which involved reversible polarization change, was achieved by Mankowsky et al. in their work on lithium niobate.^4^ Other studies^5−10^ have also explored the selective excitation of lattice vibrations under ultrashort optical or THz pulses, which are essential for achieving practical polarization switching. The absence of a predictive model poses a significant obstacle to experimentally observing ultrafast switching of electric polarization. Such a model could provide optimal pulse parameters and answer a series of questions, such as which normal mode should receive energy injection; whether energy should be injected directly into the mode that leads to switching or another strongly coupled mode; whether it is beneficial to use a series of pulses; which pulse polarization is optimal for switching; whether pulse shape affects switching; and which ferroelectric material is best suited for ultrafast switching of electric polarization, among others. In this research, we improved and tested a theoretical model for ultrafast polarization switching, which has previously been proposed in various studies.^4,11−13^ To calculate material constants of ferroelectrics as oxides and chalcogenides, first-principles methods like density functional theory (DFT) are often utilized.^14^ These methods are effective in determining the structure of stable polarized states, energy barriers, ions’ effective charges, polarization values, and the phonon spectrum.^12,15−20^ Moreover, it is important to highlight that DFT calculations’ results are highly dependent on the chosen exchange–correlation functional.^21^ Classical molecular dynamics (MD) simulations enable the examination of ultrafast polarization switching at an atomistic level^11^ and even take into account domain behavior.^22^ The proposed model aims to investigate ultrafast polarization switching in ferroelectrics. The model utilizes a system of ordinary differential equations (ODEs) to represent the time progression of the generalized coordinates within a ferroelectric material’s elementary cell. Radiation interaction is included by incorporating a perturbation force within the ODE, which functions for a specific duration. The potential energy surface (PES) is obtained from DFT calculations. Barium titanate (BTO) is used as a test material in this research as it is a well-studied, prototypical ferroelectric material. The proposed model primarily builds upon earlier works,^12,23−26^ where a similar approach was employed for polarization switching and structure changes driven by ultrashort pulses. However, two significant modifications were introduced. First, instead of representing the PES in the form of a Taylor series, we directly interpolate the PES using cubic splines. This is because switching results in substantial atomic displacement, leading to high numerical errors in the Taylor series. Second, in terms of generalized coordinates, we consider the polarization mode (*q*_p_), which undergoes the switch, and the normal mode (*Q*_IR_) where radiation is pumped. In previous studies,^12^ generalized coordinates were normal modes, while in our approach, the potential is symmetric, and we use one normal mode and other generalized coordinates for switching (for more details, please refer to ref (27)). The paper is structured as follows. In the methods section, we give details of calculating the PES and constructing the system of ODEs. The Results and Discussion section presents the data obtained for BTO, along with a discussion on metastable switching, effective friction, perturbation duration, and optimal frequency. The conclusion section provides general observations and recommendations for future experiments.

## Computational Details

**Figure 1:**
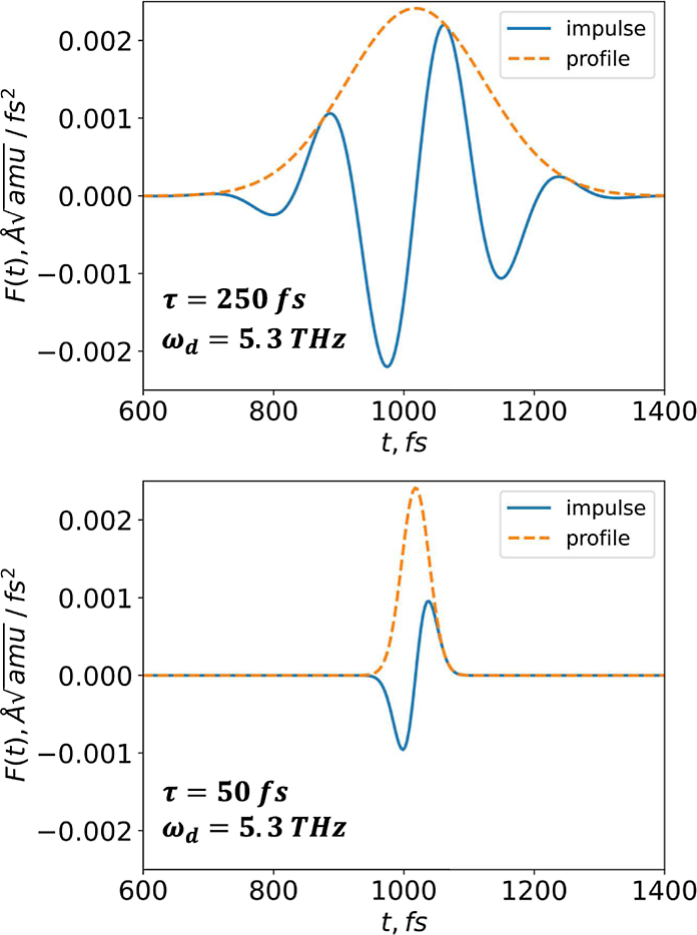
Visual representation of the perturbing force *F*(*t*) is shown for two pulse times (250 and 50 fs) and a frequency related to the high-frequency optical normal mode (5.3 THz). It is essential to note that the frequency is significantly high, allowing approximately two oscillations to fit within the 250 fs envelope.

**Figure 2:**
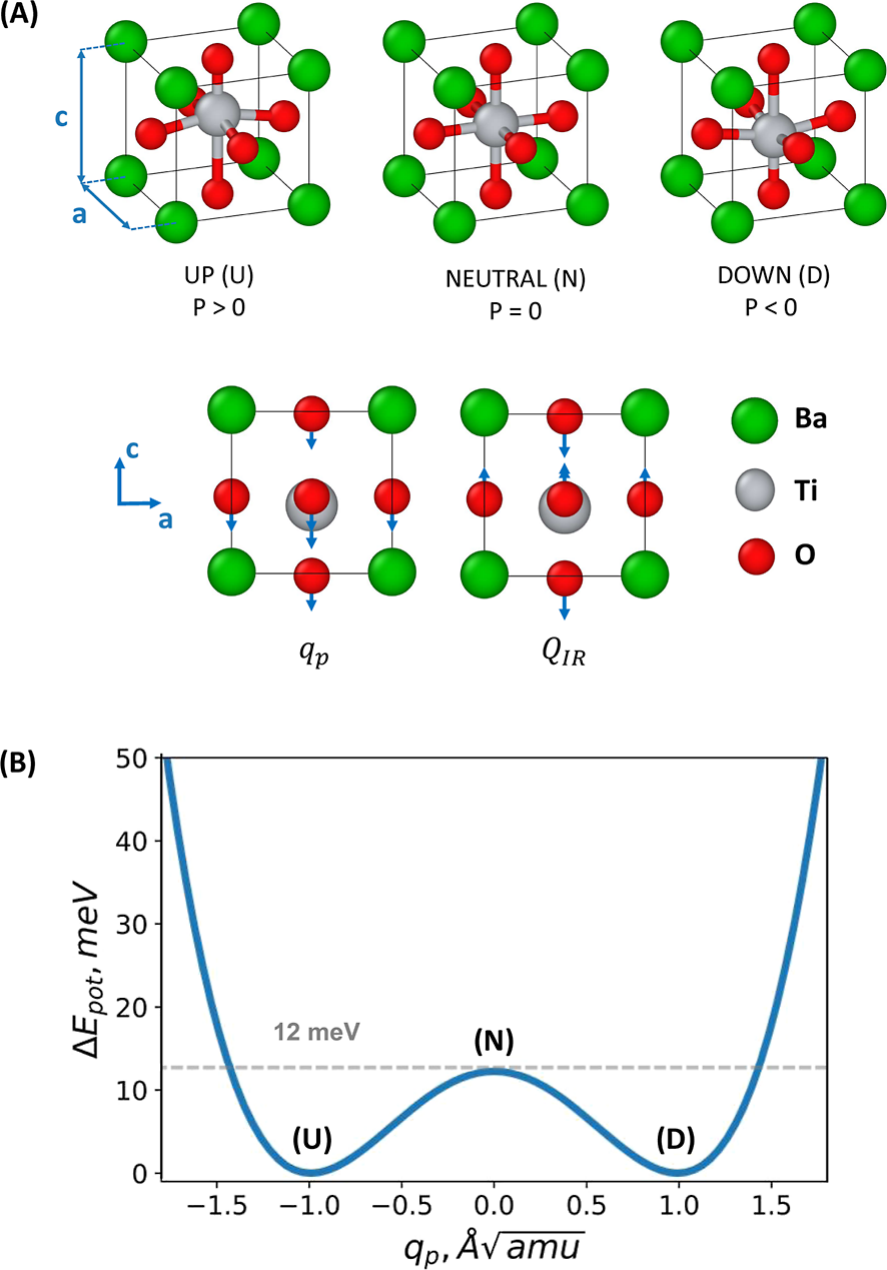
(A) Atomic illustration of the tetragonal ferroelectric phase in BTO *P*4*mm* is provided. Primarily, electric polarization switching is linked to the motion of the titanium atom along the *c*-axis: UP (U), initial polarization along the same direction to the *c*-axis; NEUTRAL (N), no polarization; DOWN (D), final polarization with opposite direction to the *c*-axis. The figure also illustrates the displacement patterns of the generalized coordinates denoted by *q*_p_ and *Q*_IR_. (B) Energy barrier for BTO divides the two stable states related to the nominal downward and upward electrical polarization. The barrier’s height, as calculated from first-principles calculations, is approximately 12 meV, which agrees well with the results from similar studies.

We take the experimental values of a material’s unit cell and relax the atomic positions to obtain the equilibrium structure. Both ionic relaxation and calculations for phonon spectra and energies were carried out using the Vienna ab initio simulation package software package,^28−31^ employing a plane-wave basis set. The projector augmented-wave pseudopotential with a general gradient approximation PBE^32^ and a cutoff energy of 600 eV is utilized in all calculations. Numerical integration over the Brillouin zone is conducted by using an 8 × 8 × 8 k-point sampling with a Γ-centered grid. The phonon dispersion curves are calculated within the framework of finite displacements (FD) using the phonopy code.^33^ All corresponding DFT calculations are executed for a perfect 2 × 2 × 2 supercell structure. After the normal modes are identified, the PES is calculated as a function of two independent normal mode generalized coordinates: *q*_p_ (polarization mode) and *Q*_IR_ (high-frequency mode). The individual atomic displacements, associated with the generalized coordinate *q*_p_, can be expressed as
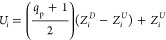
1Here, *U*_*i*_ represents the displacement of the *i*-th atom, while *Z*_*i*_^*U*^ and *Z*_*i*_^*D*^ denote the coordinates of the *i*-th atom in the direction of polarization, corresponding to equilibrium positions with positive and negative polarization along the *c*-axis, as shown in Figure 2, respectively. The individual atomic displacements, related to the generalized coordinate *Q*_IR_, are given by
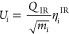
2where *U*_*i*_ is the displacement of an *i*-th atom of atomic mass *m*_*i*_ and the corresponding component of the normal mode dimensionless eigenvector η_*i*_^IR^. The PES is interpolated using cubic splines^34^ at points where DFT calculations are obtained, allowing us to define the PES continuously as *V*(*q*_p_,*Q*_IR_). The dynamic behavior of the coupled generalized coordinates is characterized by a system of associated nonlinear differential equations of motion
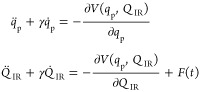
3where γ represents the effective friction coefficient, and *F*(*t*) is the initial force exerted on the system due to external pulse perturbation. The integration of eq 3 is performed using the Odeint library from the SciPy package.^34^ We assume *F*(*t*) takes the following form
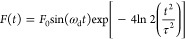
4where *F*_0_ is the force amplitude, ω_d_ is the perturbation’s driving frequency (assumed to equal ω_IR_, unless stated otherwise), and τ is the pulse’s time length. A graphical representation of how the perturbing force and its area increase with an increase in pulse duration is illustrated in Figure 1. The area of perturbing force could be resembled to the number of perturbation pulse oscillations. As *t* increases, the number of pulse oscillations also increases accordingly.

## Results and Discussion

**Figure 3:**
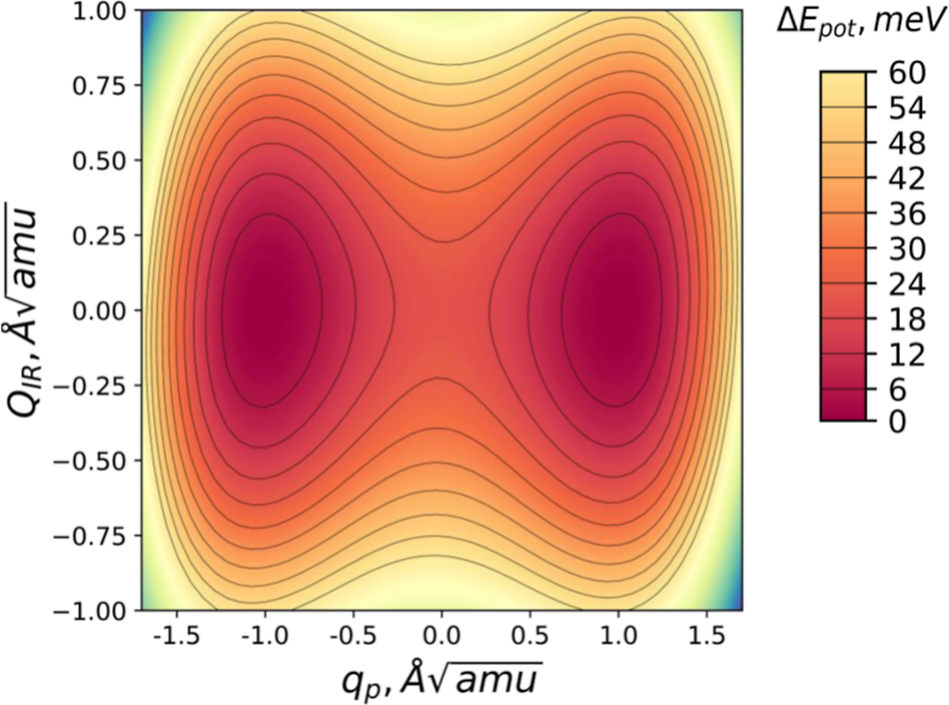
BTO PES is illustrated in generalized coordinates (*q*_p_, *Q*_IR_). The heat map displays energy in eV units, measured from the base value of the potential energy at (0, 0).

**Figure 4:**
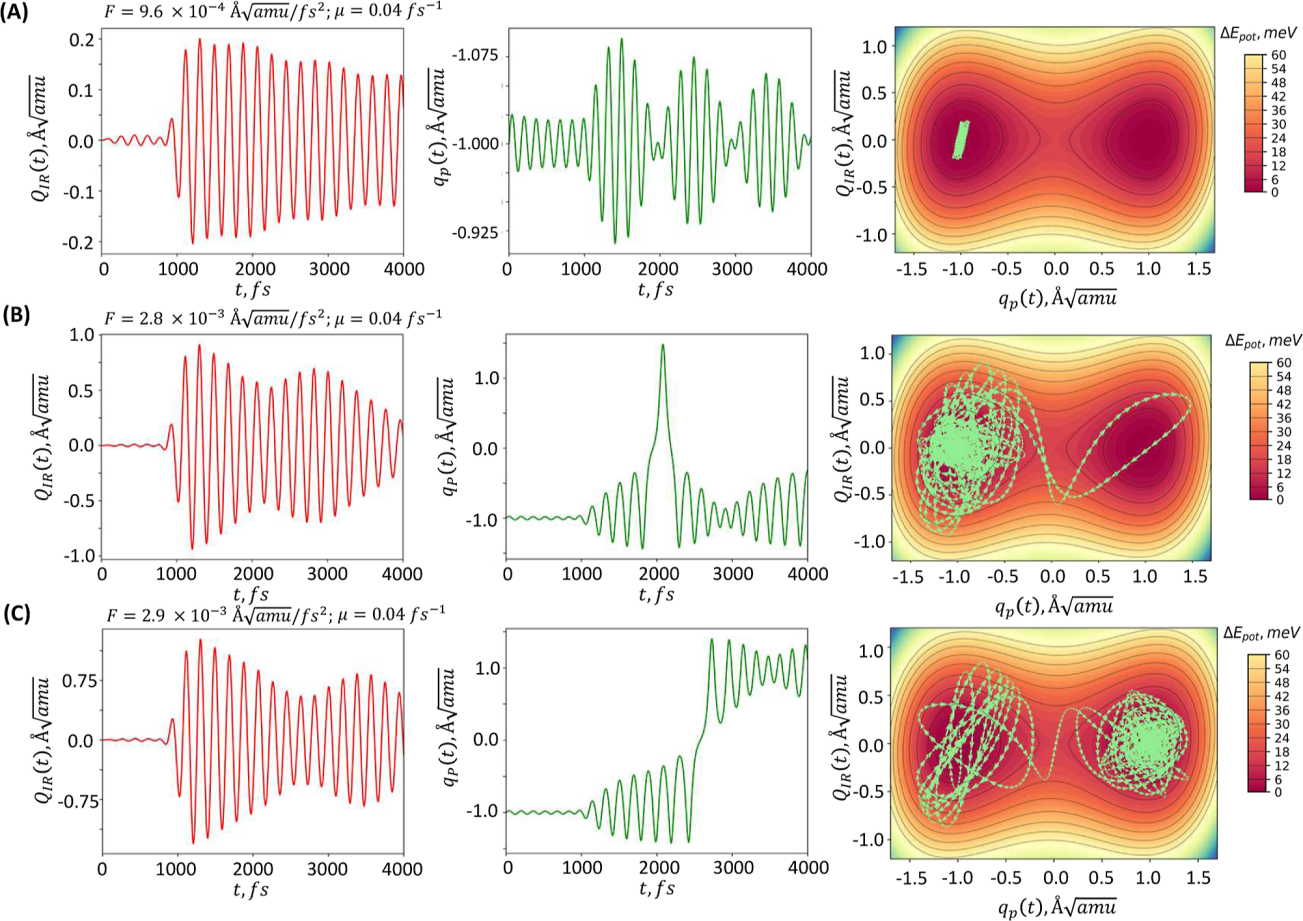
Time evolution of generalized coordinates under the influence of varying pulse amplitudes is depicted, with the trajectory of generalized coordinates on the PES shown as a green line. (A) When the perturbation amplitude is relatively small, no switching takes place and the system remains at its initial minimum (i.e., UP); (B) switching from UP to DOWN may not be “stable”—the system can momentarily enter a state with opposite (i.e., DOWN) electrical polarization, but due to inertia, it may return to and remain in the initial minimum (i.e., UP), preventing the switching from taking place; (C) if the perturbation amplitude is large enough, switching occurs, and the system transitions into a state with reversed electric polarization (i.e., DOWN) with respect to the initial one (i.e., UP). See Figure 2 to understand the difference between the UP and DOWN.

The ferroelectric state of BTO is present in the crystal structure, featuring a lattice with a *P*4*mm*(99) space group. We adopt the following experimental crystal unit cell parameters: *a* = 3.986 Å and *c* = 4.026 Å.^35^ The primitive unit cell is composed of one barium atom, one titanium atom, and three oxygen atoms (refer to Figure 2). This structure gives rise to 15 normal modes at the Γ-point, including three acoustical and 12 optical branches, which are of particular interest to us. The optical normal modes at the gamma point can be decomposed as Γ = 3*A*_1_ + *B*_1_ + 4*E*. The initial cubic symmetry *Pm*3̅*m*(221) of the paraelectric BTO crystal at 130 °C goes for transition to the ferroelectric state through atomic displacements strictly along the *c*-axis into tetragonal *P*4*mm*(99) symmetry.^36^ Consequently, the coupling between normal modes and the motion (*q*_p_) responsible for polarization switching is likely to occur with normal modes that possess large *c*-axis components in their eigenvectors. In BTO, these modes are 5, 9, and 11, corresponding to frequencies of 5.3, 8.8, and 14.1 THz. The excitation of only three low-frequency modes allowed us to avoid the nonlinear coupling between low- and high-frequency modes that is known to affect the polarization switching in such material when both are present.^37^ In this work, we chose to investigate mode 5 because it represents a typical frequency that can be achieved with modern powerful terahertz radiation sources, avoiding the presence of second harmonics.^4^ The PES was computed in the space of two generalized coordinates (*q*_p_, *Q*_IR_), with each representing the collective displacement of all atoms in the unit cell (refer to eq 1 and 2). The sampling for *q*_p_ was performed in the range from −2.0 to 2.0 with a step of 0.05 in Å , while the sampling for *Q*_IR_ was carried out in the range from −3.0 to 3.0 with a step of 0.01 in Å  units, resulting in a total of 48,000 static DFT calculations. The point representation of the PES was interpolated using cubic splines for solving the systems of ODEs. This method offers a more accurate representation of polarization switching compared to the Taylor series expansion, which is only effective in the local vicinity of the expansion point.^4,12^ A PES cross-section (shown in Figure 3) along the direction *Q*_IR_ ≈ 0 Å  enables the examination of the barrier obtained by linearly interpolating the system of atomic coordinates from an upward polarization state to a downward polarization state. For DFT calculations, the barrier height is found to be ∼12 meV, which is consistent with other calculations employing the PBE exchange–correlation potential.^21^ To analyze the trajectory of generalized coordinates under a perturbing pulse for differing perturbation amplitudes, a series of calculations was performed (see Figure 4). The effective friction coefficient was set at μ = 0.04 fs^–1^. Three distinct scenarios were observed:

1. When the perturbation force is not sufficient, the system remains at the initial minimum, with the trajectory localized nearby (see Figure 4A).
2. A scenario not typically addressed by other authors,^4,12^ but worth noting, involves the system entering a different polarization state only to return to its initial state after a period of time due to inertia. Thus, even a strong enough perturbation impulse may not alter the final electric polarization (see Figure 4B).
3. Upon reaching a specific threshold for perturbation amplitude, enough energy is transferred into the system to surpass the barrier between local minima, causing the system to switch to a state with reversed polarization (see Figure 4C).

**Figure 5:**
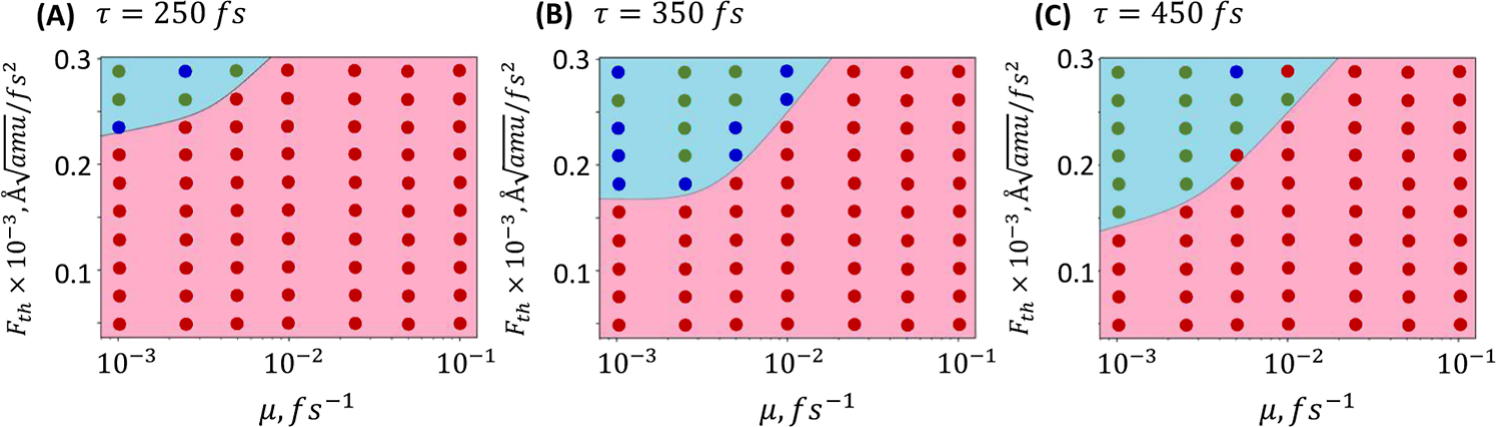
Series of computations performed for the threshold amplitude of the perturbing pulse (*F*_th_) from 0.0 to 0.3 Å /fs^2^ at three different pulse lengths: (A) 250, (B) 350, and (C) 400 fs. For each computation set, the friction coefficient (μ) was modified over a wide range of values, from 10^–3^ to 10^–1^ fs^–1^. In each calculation, the presence or absence of polarization change was noted from UP to DOWN (see Figure 2): red circles represent calculations where polarization switching did not occur; blue circles indicate instances where polarization shifted from UP to DOWN, but eventually returned to its original state; and green circles denote calculations where the polarization is stable switched to DOWN.

**Figure 6:**
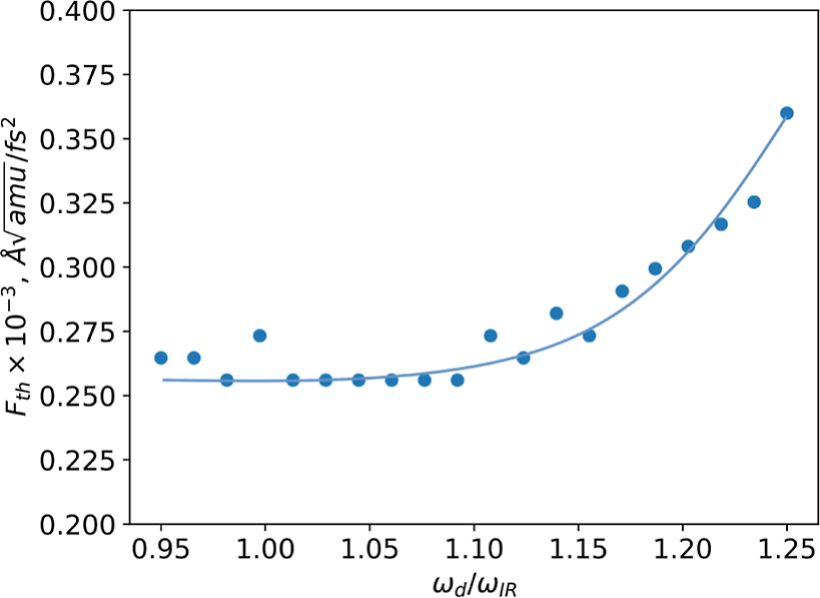
Relationship between the amplitude of the perturbing pulse, which causes switching, and the frequency of the perturbing pulse is demonstrated. The graph indicates that the lowest threshold force amplitude falls within the range of 0.95–1.10 ω_IR_. A continuous line is included merely to serve as a visual guide. Pulse duration: 250 fs.

A reversible polarization switch was previously observed in a study,^11^ where lead titanate (PTO) was modeled at the atomic level. Therefore, exposing BTO to a single polarization pulse could lead to irreversible switching if the pulse parameters fall within a narrow range. However, even with carefully chosen pulse parameters, irreversible polarization switching might not be achieved due to the chaotic nature of polarization switching.^38^ Further research is needed to investigate this hypothesis in detail. A crucial fitting parameter in the equations that describes the dynamics of generalized coordinates is the friction coefficient. This coefficient can be efficiently estimated through calibration experiments. Nonetheless, several factors can impact the friction coefficient, such as (1) the domain structure’s dependency on the geometrical dimensions of the ferroelectric material sample; (2) the influence of neighboring unit cells (not considered in this work); (3) the density of local defects. As a result, we conducted calculations by varying the friction coefficient over a broad range, analyzing its influence on the threshold switching force and switching stability (refer to Figure 5). Calculations were performed for three pulse durations: 250, 350, and 450 fs, and a set of friction coefficients ranging from 10^–3^ to 10^–1^ fs^–1^. The calculations determined whether a switch occurred and whether it was reversible or irreversible. We observed that different pulse lengths only with a friction coefficient between 10^–3^ and 10^–2^ fs generate switching of polarization. The increase in pulse length allowed us to observe a decrease in the necessary amplitude of the perturbing pulse (*F*_th_) to observe switching of polarization. Additionally, the frequency of the perturbing pulse was varied in the calculations (Figure 6). The lowest threshold amplitude was observed for frequencies in the range of 0.95–1.10 ω_IR_ for the eigenfrequency of the perturbed *Q*_IR_ mode, while the threshold force amplitude reduction was approximately 1.6 times. An analysis of the motion equation reveals that for small amplitude excitations,^23,39^ coupling effects cause renormalization of the optimal frequency, ω_IR_. A frequency shift in an underdamped oscillator is a well-studied phenomenon.^40^ Let us also estimate the fluence corresponding to the typical force at which polarization switching occurs. We adopt the smallest noted value (see Figure 6), which is on the order of *F*_th_ = 2.5 × 10^–4^ Å . This force (*F*_th_) is equivalent to the acceleration  Å /fs^2^, which represents the acceleration of the Ba^+^ ion created by the electric field . Subsequently, the energy density of such a field is linked to fluence *W* = ϵ_0_*E*^2^/2·Ω = *F*_th_·*S*, which infers *F*_th_ = ϵ_0_·*E*^2^/2·*h*, where Ω, *S*, and *h* represent the volume, surface area, and the length of the unit cell in the “c” direction (see Figure 2a), respectively, and ϵ_0_ is the vacuum permittivity. This simple estimation yields a value of *F*_th_ = 250 mJ/cm^2^. Although this is a rather basic analysis, the derived estimation should be approached with caution. For comparison, in the article^4^ studying lithium niobate, the onset of polarization switching occurred at fluences of 95 mJ/cm^2^, which is on the same order of magnitude as the estimated *F*_th_ for BTO.

## Conclusions

In this study, a model was examined and evaluated to characterize the ultrafast switching polarization in ferroelectric materials using BTO as the test case. Analyzing the proposed model indicates that an operative range of the friction coefficient exists where the ultrafast switching polarization has the highest probability of happening. Such probability increases with the increasing pulse, and the smallest threshold force amplitude necessary for switching is achieved within the range of 0.95–1.10 ω_IR_, where ω_IR_ represents the normal-mode frequency. Polarization switching has been shown to be reversible, and it is probably a random process, meaning that slight changes in the perturbing pulse parameters might lead to an opposite (i.e., DOWN) final polarization with respect to the initial one (i.e., UP). See Figure 2 to understand the meaning of UP and DOWN. Thus, the complexity of the model in the future should include arbitrary polarization of the perturbing pulse, which may prove difficult to interpret, and the possibility of considering multipulse cases. For example, involving the depolarization potential, which is generated by secondary high-frequency pulses, which inject energy into the electronic subsystem, raises the electronic temperature to tens of *eV*, favoring the switching of polarization as seen in previous works.^4,41,42^
